# Impact of stochastic fluctuations in the cell free layer on nitric oxide bioavailability

**DOI:** 10.3389/fncom.2015.00131

**Published:** 2015-10-27

**Authors:** Sang-Woo Park, Marcos Intaglietta, Daniel M. Tartakovsky

**Affiliations:** ^1^Department of Mechanical and Aerospace Engineering, University of CaliforniaSan Diego, La Jolla, CA, USA; ^2^Bioengineering Department, University of CaliforniaSan Diego, La Jolla, CA, USA

**Keywords:** microcirculation, wall shear stress, stochastic, nitric oxide, endothelium

## Abstract

A plasma stratum (cell free layer or CFL) generated by flowing blood interposed between the red blood cell (RBC) core and the endothelium affects generation, consumption, and transport of nitric oxide (NO) in the microcirculation. CFL width is a principal factor modulating NO diffusion and vessel wall shears stress development, thus significantly affecting NO bioavailability. Since the CFL is bounded by the surface formed by the chaotically moving RBCs and the stationary but spatially non-uniform endothelial surface, its width fluctuates randomly in time and space. We analyze how these stochastic fluctuations affect NO transport in the CFL and NO bioavailability. We show that effects due to random boundaries do not average to zero and lead to an increase of NO bioavailability. Since endothelial production of NO is significantly enhanced by temporal variability of wall shear stress, we posit that stochastic shear stress stimulation of the endothelium yields the baseline continual production of NO by the endothelium. The proposed stochastic formulation captures the natural continuous and microscopic variability, whose amplitude is measurable and is of the scale of cellular dimensions. It provides a realistic model of NO generation and regulation.

## 1. Introduction

Nitric oxide (NO) plays a critical role in the local control of smooth muscle tone and the regulation of blood flow at the microvascular level. Its distribution in the microcirculation is determined by the balance between NO production and consumption in the blood and tissue compartments. The local concentration of NO in blood results from the competition between NO diffusing from the endothelium and NO scavenging by hemoglobin in red blood cells (RBCs) or at times dissolved in plasma. Mathematical modeling was used (Vaughn et al., 1998a,b; Condorelli and George, 2002; Kavdia and Popel, 2003; Lamkin-Kennard et al., 2004; Chen et al., 2006; Ong et al., 2011b; Sriram et al., 2011) to determine the NO distribution in blood vessels simulated by cylindrical and parallel-plate compartments, as a function of local transport parameters such as NO production rate, scavenging reaction rate and diffusion coefficients in blood and tissue. All these analyses assume deterministic boundary conditions.

Mechanotransduction generates a significant portion of the NO involved in the regulation of blood flow (Condorelli and George, 2002; Chen et al., 2006). This mechanical effector links the biochemistry of NO production by the endothelium with shear stress induced by blood flow on the vascular wall (WSS). This coupling is tight because NO bioavailability in the vascular wall determines vessel diameter, the anatomical component of vascular flow resistance. WSS is generated at the vascular wall by tangential stresses caused by flowing blood whose RBC concentration (hematocrit or Hct) diminishes from maximal at the blood flow core to zero in the cell free layer (CFL) adjacent to the vessel wall.

Blood in microcirculation can be modeled as a two-layer system consisting of two immiscible fluids. Flow velocity profiles are parabolic within the CFL and plug-like in the RBC core region (Sharan et al., 1997; Martini et al., 2005; Sriram et al., 2011) where the fluid exhibits non-Newtonian behavior. Such models provide a coarse-grained description of more detailed simulations of variations of the WSS induced by a passing column of RBCs (e.g., Dupin et al., 2007). Changes of NO production due to changes in WSS caused by the variation of blood flow and plasma viscosity (Tsai et al., 1998) appear to dominate other factors, such as changes in hemoglobin concentration or Hct (Kavdia and Popel, 2003; Lamkin-Kennard et al., 2004; Chen et al., 2006; Namgung et al., 2011; Sriram et al., 2011). Experimental evidence (e.g., Kanai et al., 1995) suggests that NO production rate by the endothelium varies linearly with WSS (see also Andrews et al., 2010; Ong et al., 2011b).

However, there is a fundamental difference in the rate of NO production between steady and time-varying WSS (Frangos et al., 1996; Ong et al., 2011b), the latter being significantly greater. Temporal variability arises from action of the heart and the respiratory cycle which although attenuated is present in the microcirculation, or vasomotion which is rooted in the microcirculation.

Endothelial response to shear stress is mostly studied in parallel plate flow chambers, where endothelial cells grow to confluent layers. However, it is generally recognized that this experimental setup differs from *in vivo* conditions, since it relies on perfusion with cell culture media fluid instead of blood. (We are aware of a study by Yalcin et al., 2008 that did use RBC suspension, but it did not compare results with incubation medium). Blood perfusion introduces other, hitherto not considered, sources of temporal variability due to the microscopic spatial fluctuations of the CFL width. Spatial variability is generated by blood flowing over the spatially non-uniform endothelial surface (Barbee et al., 1994; Sato et al., 2000). Spatial and temporal variability is generated at the blood boundary of the CFL due to the changes in motion, position and shape of RBCs in the outer layer of the flowing blood column. As a consequence of these phenomena fluid in the CFL flows between a spatially variable endothelial boundary and spatially and temporally highly irregular RBC surface (Kim et al., 2006, 2007), both of which randomly affect WSS. These effects are non-existent in the absence of RBCs. Furthermore, as a consequence of their stochastic nature they potentially include all forms of variability. The frequency of stochastic fluctuations of the WSS due to spatio-temporal variability of the CFL may significantly increase the bioavailability of NO. Evidence for this hypothesis is provided by an observation that exposing the tissue damaged by ischemia reperfusion to diagnostic ultrasound improves microvascular functionality, an effect that is significantly reduced by administration of L-NAME (Hightower and Intaglietta, 2008).

These unpredictable fluctuations can be analyzed by treating the CFL width at any given location as a random field that determines the distance between the NO source and its sink. The stochasticity of the CFL width also affects NO concentration due to its repercussion on the local, microscopic variability of the flow field, and therefore on shear rates and shear stresses. While the random spatio-temporal fluctuations of CFL width and its bounding surfaces clearly affect NO bioavailability and production rate, most studies (including those mentioned above) treat the interface between the RBC column and plasma as a smooth deterministic surface. In the present study, we adopt a more realistic approach by treating the surface between flowing RBCs and the CFL as a random field whose statistics are obtained from experimental studies (Ong et al., 2011a,b). Within this conceptual framework, we formulate a model that determines the distribution of NO concentration in the region of the interface between blood and tissue at the blood vessel wall (RBC-rich core, cell free layer, and tissue layer) (Vaughn et al., 1998a).

## 2. Mathematical model of NO transport

### 2.1. Model formulation

Our analysis deals with NO transport in the geometrical configuration associated with the standard Krogh tissue cylinder model. We consider an arteriolar cross-section that consists of the RBC-rich core ($E^{1}:0\leq r\leq r_{1}$), the CFL ($E^{2}:r_{1}<r\leq r_{2}$), the endothelial-cell region ($E^{3}:r_{2}<r\leq r_{3}$), and the smooth-muscle region ($E^{4}:r_{3}<r\leq r_{4}$). Stochastic fluctuations of the interface formed by flowing RBCs, *r*_1_(θ, *t*), are modeled by treating it as a random function of both angular coordinate θ and time *t*, i.e., *r*_1_ = *r*_1_(θ, *t*; ω) with ω ∈ Ω indicating a realization (“coordinate”) in the probability space Ω. This renders the CFL width *w* = *r*_2_ − *r*_1_ random, i.e., *w* = *w*(θ, *t*; ω). Our goal is to capture the effects of stochastic fluctuations of the RBC-CFL interface *r*_1_(θ, *t*; ω) on distribution of NO concentration, *C*_NO_, in the Krogh tissue cylinder D = {(*r*, θ):0 ≤ *r* ≤ *r*_4_, 0 ≤ θ ≤ 2π}.

In each region of the computational domain, *E*^*i*^ (*i* = 1, …, 4), the concentration *C*_NO_ satisfies a reaction-diffusion equation

(1) $$\begin{array}{ll} \frac{\partial C_{NO}}{\partial t}=D_{i}\nabla^{2}C_{NO}-k_{i}C_{NO},\;\left(r,\theta \right)\in E^{i}, & \; \end{array}$$

where *D*_*i*_ and *k*_*i*_ are the diffusion coefficient and degradation (reaction) rate in the *i*-th region, respectively. These four equations are coupled by the continuity conditions at the interfaces *r*_*i*_ (*i* = 1…, 3),

(2) $$\begin{array}{ll} C_{NO}^{-}=C_{NO}^{+},\;F_{n}^{-}-F_{n}^{+}={\dot{q}}_{i},\;r=r_{i}. & \; \end{array}$$

Here the superscripts ^−^ and ^+^ indicate the left and right limits of the corresponding quantities at the *i*-th interface, *F*_*n*_ = **F**_*i*_ · **n**_*i*_ is the normal component of Fick's flux **F**_*i*_ = −*D*_*i*_∇*C*_NO_ at the *i*-th interface whose outward unit normal is **n**_*i*_, and ${\dot{q}}_{i}$ denotes the NO production rates at the interface *r* = *r*_*i*_. Since only endothelium cells are involved in NO production, ${\dot{q}}_{1}\equiv 0$. We assume that no nitric oxide leaves the outer boundary of the smooth-muscle region, *r* = *r*_4_, so that

(3) $$\begin{array}{ll} \text{n}\cdot \nabla C=0,\;r=r_{4}. & \; \end{array}$$

The coupling of the reaction-diffusion Equations (1) at the interfaces *r* = *r*_*i*_ (*i* = 1, 2, 3) propagates uncertainty (randomness) in the topology of the RBC-CFL interface *r*_1_(θ, *t*; ω) through the modeling process, leading to randomly varying NO concentration *C*_NO_(*r*, θ, *t*; ω) throughout the Krogh tissue cylinder.

The problem formulation given by Equations (1)–(3) implicitly assumes that blood flow is laminar, fully-developed, and incompressible, vessel walls are impermeable to blood flow, NO concentration at the vessel inlet equals that at the vessel outlet, and all the reaction rates are spatially uniform. In the deterministic setting with a uniform CFL width, these assumptions imply that the radial (*u*_*r*_) and angular (*u*_θ_) components of flow velocity $\text{u}={\left(u_{r},u_{\theta },V\right)}^{⊤}$ are *u*_*r*_ = *u*_θ_ ≡ 0, its longitudinal component is *V* = *V*(*r*), and NO concentration *C*_NO_ = *C*_NO_(*r*). This results in advective flux of NO in the blood vessel, **u** · ∇*C*_NO_, that is identically zero. In the stochastic setting with randomly fluctuating CFL width, advective flux ***u***·∇*C*_NO_ is zero in the mean (Tartakovsky and Xiu, 2006; Park et al., 2012).

### 2.2. Model parameterization

While the reaction rates *k*_*i*_ in the endothelium (*i* = 3) and tissue (*i* = 4) can be considered constant, the reaction rate in the RBC-rich core (*k*_1_) is related to hemoglobin levels. The latter depends on hematocrit *H*(*r*) and radial component of blood flow velocity *V*(*r*, θ, *t*). Let *k*_*s*_ denote a reference rate of NO scavenging by RBCs at a reference level of hematocrit *H*_*s*_. Then, the NO scavenging rate *k*_1_ corresponding to a given hematocrit level *H*_*c*_ is given by Ong et al. (2011b) and Chen et al. (2006)

(4) $$\begin{array}{ll} k_{1}=\frac{H_{c}}{H_{s}}k_{s}. & \; \end{array}$$

The hematocrit ratio *H*_*c*_/*H*_*s*_ is determined by mass conservation,

(5) $$\begin{array}{ll} \overset{2\pi }{\underset{0}{\int }}\overset{r_{2}}{\underset{0}{\int }}H\left(r,t\right)V\left(r,\theta ,t\right)rdrd\theta =H_{s}\overset{2\pi }{\underset{0}{\int }}\overset{r_{2}}{\underset{0}{\int }}V\left(r,\theta ,t\right)rdrd\theta . & \; \end{array}$$

In the general stochastic framework we advocate here, blood is a two-phase fluid that exhibits non-Newtonian behavior in the RBC-rich core and Newtonian one in the CFL, with the random surface *r*_1_(θ, *t*; ω) separating the two regions. This implies that flow velocity *V*(*r*, θ, *t*; ω) is random as well, being given by a solution of corresponding flow equations in random domains (Park et al., 2012). To focus on NO transport, we simplify the flow calculations by adopting two alternative approximations.

The first is based on a lubrication approximation in which random geometry parameterizes an otherwise deterministic velocity profile (Tartakovsky and Xiu, 2006). This approach yields a random velocity profile *V*(*r*, θ, *t*; ω),

(6) $$\begin{array}{l} \frac{V}{V_{\text{max}}}=\{\begin{array}{ll} 1-\frac{\mu_{p}}{\mu_{c}}\frac{r^{2}}{r_{2}^{2}}-\left(1-\frac{\mu_{p}}{\mu_{c}}\right)\frac{r_{1}^{2}}{r_{2}^{2}} & \;0\leq r\leq r_{1}\\ 1-\frac{r^{2}}{r_{2}^{2}} & \;r_{1}\leq r\leq r_{2} \end{array} \end{array}$$

where $V_{max}=Jr_{2}^{2}∕\left(4\mu_{p}\right)$ is the (maximum) centerline velocity, *J* is the externally imposed pressure gradient, and μ_*p*_ and μ_*c*_ are the viscosities of the plasma and RBC-rich core. In this formulation, the only source of the non-Newtonian behavior of the RBC-rich core is the dependence of the core viscosity μ_*c*_ on the (random) CFL width. Following Martini et al. (2005) and many others, we assume a linear relationship μ_*c*_ = 0.1678*H*_*c*_ − 4.348 between a hematocrit level *H*_*c*_ and the viscosity of the RBC core μ_*c*_. Specifying a (random) radial distribution of hematocrit, *H* = *H*(*r, t*; ω), as a step function

(7) $$\frac{H}{H_{c}}=\{\begin{array}{ll} 1 & \;0\leq r\leq r_{1}\\ 0 & \;r_{1}<r\leq r_{2} \end{array}$$

enables one to compute the randomly fluctuating NO scavenging rate *k*_1_(*t*; ω) by combining Equations (4)–(7). First, the system of Equations (5)–(7) was solved using Matlab function “solve” to compute *H*_*c*_ for μ_*p*_ = 1.2 cP and two values of *H*_*s*_. Then *k*_1_(*t*; ω) was obtained from Equation (4).

The second alternative for obtaining *k*_1_(*t*; ω) treats blood as a single-phase fluid with a parabolic velocity profile

(8) $$\begin{array}{ll} \frac{V}{V_{max}}=1-\frac{r^{2}}{r_{2}^{2}},\;0\leq r\leq r_{2}. & \; \end{array}$$

This formulation replaces the CFL and the random RBC-CFL interface *r*_1_(θ, *t*; ω) with a radial distribution of hematocrit,

(9) $$\frac{H}{H_{c}}=\{\begin{array}{ll} 1 & \;0\leq r\leq r_{1}\\ {\left(\frac{r_{2}-r}{r_{2}-r_{1}}\right)}^{2} & \;r_{1}\leq r\leq r_{2} \end{array}.$$

Substituting Equations (8) and (9) into Equations (4) and (5) yields an alternative expression for the NO scavenging rate *k*_1_(*t*; ω). This approach was used by Ong et al. (2011a) in the deterministic context that treated *r*_1_(θ, *t*) as constant.

Finally, we allow the NO production rates by the endothelium, i.e., ${\dot{q}}_{2}$ and ${\dot{q}}_{3}$ in Equation (2), to vary with the wall shear stress τ_*w*_ exerted on the endothelium walls by blood flow. Following Ong et al. (2011a,b), Vaughn et al. (1998a) and others, we assume a linear relation

(10) $$\begin{array}{ll} {\dot{q}}_{2}={\dot{q}}_{3}=\frac{\tau_{w}}{\tau_{w}^{⋆}}{\dot{q}}^{⋆},\;\tau_{w}=\mu_{p}\frac{V_{e}}{w}, & \; \end{array}$$

where $\tau_{w}^{⋆}$ is the reference wall shear stress, ${\dot{q}}^{⋆}$ is the control NO production rate, and *V*_*e*_ is the mean velocity at the outer edge of the RBC core. These production rates fluctuate randomly, i.e., ${\dot{q}}_{2}\left(t;\omega \right)$ and ${\dot{q}}_{3}\left(t;\omega \right)$, due to their dependence on the random flow velocity *V* and the CFL width *w*(θ, *t*; ω) = *r*_2_ − *r*_1_(θ, *t*; ω).

In the numerical results reported below we assume the diffusion coefficients *D*_*i*_ in Equation (1) to be the same and equal to *D*. Its value and the values of the remaining parameters used in our are model are reported in Table 1.

**Table 1:** **Model parameters and their values**.

**Parameter**	**Symbol**	**Value**	**Units**	**Source**
Vessel radius	*r*_2_	23.3	μm	Ong et al., 2011a
Blood lumen width	$r_{1}={\bar{r}}_{1}+r^{\prime }$	random	μm	–
Mean cell free layer width	$\bar{w}=r_{2}-{\bar{r}}_{1}$	2.73 or 3.22	μm	Ong et al., 2011a
Endothelial cell thickness	*r*_3_ − *r*_2_	2.5	μm	Kuo et al., 1990
Tissue layer thickness	*r*(∞)−*r*_3_	2500.0	μm	–
Diffusion coefficient	*D*	3300.0	μm^2^/s	Vaughn et al., 1998b
Control NO production rate	${\dot{q}}_{NO}^{⋆}$	2.65 ·10^−14^	μmol/(μm^2^s)	Vaughn et al., 1998b
NO scavenging rate at Hc 40%	*k*_*sys*_	382.5	1/s	Chen et al., 2006
NO scavenging rate in endothelium	*k*_*EC*_	0.1	1/s	Lamkin-Kennard et al., 2004
NO scavenging rate in tissue	*k*_*T*_	0.1	1/s	Lamkin-Kennard et al., 2004
Plasma viscosity	μ_*p*_	1.2	cP	Zhang et al., 2009
Reference wall shear stress	τ_*w, ref*_	2.4	Pa	Kavdia and Popel, 2003

We represent spatio-temporal variations of the RBC-CFL interface,

(11) $$\begin{array}{ll} r_{1}\left(\theta ,t;\omega \right)=\left[{\bar{r}}_{t}+r_{t}^{\prime }\left(t;\omega \right)\right]\left[{\bar{r}}_{\theta }+r_{\theta }^{\prime }\left(\theta ;\omega \right)\right], & \; \end{array}$$

as the product of mutually uncorrelated temporal and angular fluctuations *r*_*t*_(*t*; ω) and *r*_θ_(θ; ω), respectively. A Reynolds decomposition is used to represent each of these fields, $r=\bar{r}+r^{\prime }$, as the sum of its ensemble mean $\bar{r}$ and zero-mean fluctuations *r*′. Setting ${\bar{r}}_{\theta }=1$ yields the mean and variance of the RBC-CFL interface: ${\bar{r}}_{1}={\bar{r}}_{t}$ and $\sigma_{r}^{2}={\bar{r}}_{t}^{2}\sigma_{\theta }^{2}+\sigma_{t}^{2}\left(1+\sigma_{\theta }^{2}\right)$. The coefficient of variation of the CFL width, $CV_{w}=\sigma_{w}∕\bar{w}$, is given by

(12) $$\begin{array}{ll} CV_{w}^{2}={\left(\frac{{\bar{r}}_{t}}{\bar{w}}\right)}^{2}\sigma_{\theta }^{2}+CV_{t}^{2}\left(1+\sigma_{\theta }^{2}\right) & \; \end{array}$$

where $\bar{w}=r_{2}-{\bar{r}}_{1}$ is the mean CFL width and σ_*w*_ is its standard deviation.

Since the random field $r_{\theta }^{\prime }\left(\theta ,\omega \right)$ is periodic, a truncated Fourier-type expansion

(13) $$\begin{array}{ll} r_{\theta }^{\prime }\left(\theta ;\omega \right)\approx \sigma_{\theta }\overset{N_{\theta }}{\underset{n=-N_{\theta }}{\sum }}\nu_{n}\left(\omega \right)e^{-in\theta } & \; \end{array}$$

provides its natural representation. Here the eigenvalues ν_*n*_(ω) are complex zero-mean random variables, whose real and imaginary parts are mutually independent for all *n*. Each has zero mean and variance $\sigma_{n}^{2}=C_{n}∕4$, where

(14) $$\begin{array}{ll} C_{n}=\frac{1}{\pi }\overset{2\pi }{\underset{0}{\int }}C_{\theta }^{p}\cos\left(n\theta \right)d\theta ,\;-N\leq n\leq N & \; \end{array}$$

are coefficients of the Fourier cosine expansion of a 2π-periodic covariance function $C_{\theta }^{p}$ of the random field $r_{\theta }^{\prime }\left(\theta ,\omega \right)$. It is constructed as follows. First, we note that statistics of $r_{\theta }^{\prime }\left(\theta ;\omega \right)$ are rotationally invariant on the circle, such that a covariance function *C*_θ_ is

(15) $$\langle r_{\theta }^{\prime }(\theta_{1};\omega )r_{\theta }^{\prime }(\theta_{2};\omega )\rangle =C_{\theta }(\Delta_{\theta }),\;\Delta_{\theta }=\;\left|\theta_{1}-\theta_{2}\right|.$$

Then $C_{\theta }^{p}$ is constructed by extending the covariance function *C*_θ_ of the random field $r_{\theta }^{\prime }\left(\theta ,\omega \right)$ to a 2π-periodic periodic domain. We employ a Gaussian covariance function $C_{\theta }\left(\Delta_{\theta }\right)=\exp\left(-\Delta_{\theta }^{2}∕l_{\theta }^{2}\right)$ with the correlation length *l*_θ_. The decay of the Fourier cosine coefficients *C*_*n*_ determines the number of terms *N*_θ_ in the expansion in Equation (13) that is required to achieve a given truncation error. As the correlation length *l*_θ_ decreases, *N*_θ_ increases.

We represent the random field $r_{t}^{\prime }\left(t;\omega \right)$ via a truncated Karhunen-Loéve expansion,

(16) $$\begin{array}{ll} r_{t}^{\prime }\left(t,\omega \right)=\sigma_{t}\overset{N_{t}}{\underset{m=1}{\sum }}\sqrt{\lambda_{m}}f_{m}\left(t\right)Y_{m}\left(\omega \right), & \; \end{array}$$

where *Y*_*m*_(ω) (*m* ≥ 1) are independent random variables, and λ_*m*_ and *f*_*m*_(*t*) are, respectively, the eigenvalues and eigenfunctions of Fredholm equations,

(17) $$\begin{array}{ll} \overset{T}{\underset{0}{\int }}\rho_{t}\left(t,t^{\prime }\right)f_{m}\left(t^{\prime }\right)dt^{\prime }=\lambda_{m}f_{m}\left(t\right),\;m\geq 1. & \; \end{array}$$

For an exponential correlation function $\rho_{t}\left(t,t^{\prime }\right)=\exp\left(-|t-t^{\prime }|∕l_{t}\right)$ with the correlation length *l*_*t*_ > 0, the eigenvalue problems in Equation (17) admit an analytical solution (Lin et al., 2010),

(18) $$\begin{array}{ll} \lambda_{m}=\frac{2l_{t}}{l_{t}^{2}\gamma_{m}^{2}+1},\;f_{m}=\frac{l_{t}\gamma_{m}\cos\left(\gamma_{m}t\right)+\sin\left(\gamma_{m}t\right)}{\sqrt{\left(l_{t}^{2}\gamma_{m}^{2}+1\right)T∕2+l_{t}}} & \; \end{array}$$

where γ_*m*_ are solutions of $\left(l_{t}^{2}\gamma^{2}-1\right)\sin\left(\gamma T\right)=2l_{t}\gamma \cos\left(\gamma T\right)$ and *m* ≥ 1. The truncation error of the Karhunen-Loéve expansion in Equation (16) depends on the correlation length *l*_*t*_. The smaller the correlation length, the more terms *N*_*t*_ are necessary to represent the random field *r*′(*t*, ω) with a given degree of accuracy.

Within the statistical framework adopted here, the random RBC-CFL interface is characterized by four parameters: variances $\sigma_{\theta }^{2}$ and $\sigma_{t}^{2}$, and correlation lengths *l*_θ_ and *l*_*t*_. Experimental data, such as those reported by Kim et al. (2007), can be used to estimate these statistics. Table 2 contains the values of these parameters used in our simulations.

**Table 2:** **Statistical parameters and summary of simulation results**.

**Case**	***D*_*t*_**	***D*_θ_**	**σ_*t*_**	**σ_θ_**	***l*_*t*_**	***l*_θ_**	**CV_*w*_**	**R**	**$\bar{\omega }$ (μm)**
Temporal variation	39		0.15		0.008		0.327	5.6	3.22
	39		0.203		0.008		0.442	12.7	3.22
	39		0.24		0.008		0.523	20.8	3.22
	45		0.12		0.007		0.309	5.2	2.73
	45		0.15		0.007		0.386	9.3	2.73
	45		0.172		0.007		0.443	13.7	2.73
	45		0.2		0.007		0.515	34.0	2.73
Temporal and spatial variation	39	6	0.203	0.07	0.008	1	0.515	16.2	3.22
	39	6	0.203	0.1	0.008	1	0.582	22.0	3.22
	39	9	0.203	0.07	0.008	0.6	0.543	18.1	3.22
	39	9	0.15	0.07	0.008	0.5	0.446	10.7	3.22
	39	9	0.15	0.1	0.008	0.5	0.543	19.9	3.22
	39	9	0.15	0.12	0.008	0.5	0.615	33.1	3.22
	45	9	0.12	0.07	0.007	0.5	0.432	10.6	2.73
	45	9	0.12	0.1	0.007	0.5	0.531	20.3	2.73
	45	9	0.12	0.12	0.007	0.5	0.603	34.0	2.73

### 2.3. Numerical solution

#### 2.3.1. Mapping onto deterministic domain

We introduce a new coordinate system (ξ_1_, ξ_2_), which maps the original stochastic domain D onto a rectangle *B* = {(ξ_1_, ξ_2_):−1 ≤ ξ_1_ ≤ 1, 0 ≤ ξ_2_ ≤ 2π}. A mapping $D=\cup_{i=1}^{4}E^{i}\to B$ is accomplished analytically by the coordinate transformation

(19) $$\begin{array}{ll} r=r_{i-1}\;+\;\frac{\xi_{1}+1}{2}\left(r_{i}\;-\;r_{i-1}\right),\;\theta \;=\;\xi_{2};\;\left(r,\theta \right)\in E^{i} & \; \end{array}$$

where *i* = 1, …, 4 and *r*_0_ = 0. The random RBC-CFL interface *r*_1_(θ, *t*; ω) is represented by the expansions described above.

#### 2.3.2. Transformed stochastic equations

The mapping defined by Equation (19) renders the transformation Jacobian

(20) $$\begin{array}{ll} J\left(\xi_{1},\xi_{2},\omega \right)\equiv \frac{\partial \left(r,\theta \right)}{\partial \left(\xi_{1},\xi_{2}\right)}\;=\;J\left[\xi_{1},\xi_{2},Y_{1}\left(\omega \right),\cdots \;,Y_{K}\left(\omega \right)\right]. & \; \end{array}$$

and other related metrics stochastic, i.e., dependent on a set of *K* = 2*N*_θ_ + *N*_*t*_ independent random variables ${\left\{Y_{i}\left(\omega \right)\right\}}_{i=1}^{K}$. The first *N*_*t*_ variables *Y*_1_, …, *Y*_*N*_*t*__ coincide with those introduced in Equation (16) and the remaining 2*N*_θ_ variables represent their counterparts in Equation (13), such that *Y*_*N*_*t*_ + 1_ = ν_−*N*_θ__, …, *Y*_*K*_ = ν_*N*_θ__. Consequently, the deterministic reaction-diffusion Equations (1) are transformed into stochastic equations of the form (Appendix)

(21) $$\begin{array}{l} \frac{\partial C_{NO}}{\partial t}=\overset{2}{\underset{i,j=1}{\sum }}\frac{\partial }{\partial \xi_{i}}(DA_{ij}\frac{\partial C_{NO}}{\partial \xi_{j}})-kC_{NO} \end{array}$$

where the random coefficients *A*_11_, *A*_12_ = *A*_21_, and *A*_22_ are given by Equation (A2) in the Appendix.

These stochastic differential equations on the deterministic domain *B* can be solved with a variety of well-established techniques, including perturbation-based moment equations (Tartakovsky and Winter, 2001), stochastic finite elements (Ghanem and Spanos, 1991), and stochastic collocation on sparse grids (Lin et al., 2010, and the references therein). In the subsequent numerical simulations we employ the latter approach (Appendix).

## 3. Results

Solutions of the stochastic system of transport Equations (1)–(3) are given in terms of their statistical moments. Ensemble means, e.g., mean NO concentration ${\bar{C}}_{NO}$, serve as unbiased predictors of the system behavior; variances, e.g., NO concentration variance $\sigma_{C}^{2}$, act as a measure of predictive uncertainty.

### 3.1. Data-driven model parameterization

**Figure 1:**
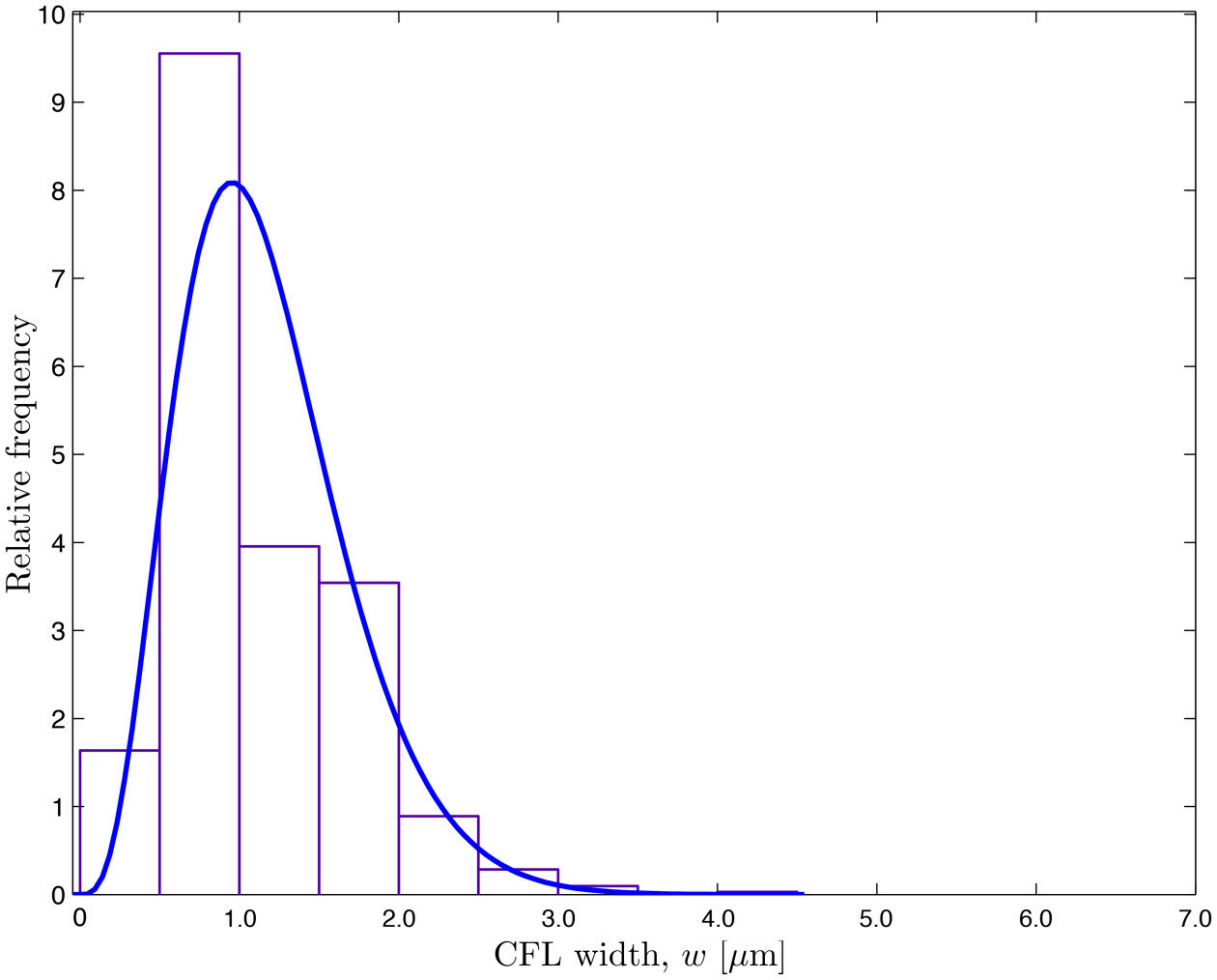
**Frequency distribution of the temporally fluctuating CFL width ***w*** reported by Ong et al. (2011a) and the fitted β-distribution**.

**Figure 2:**
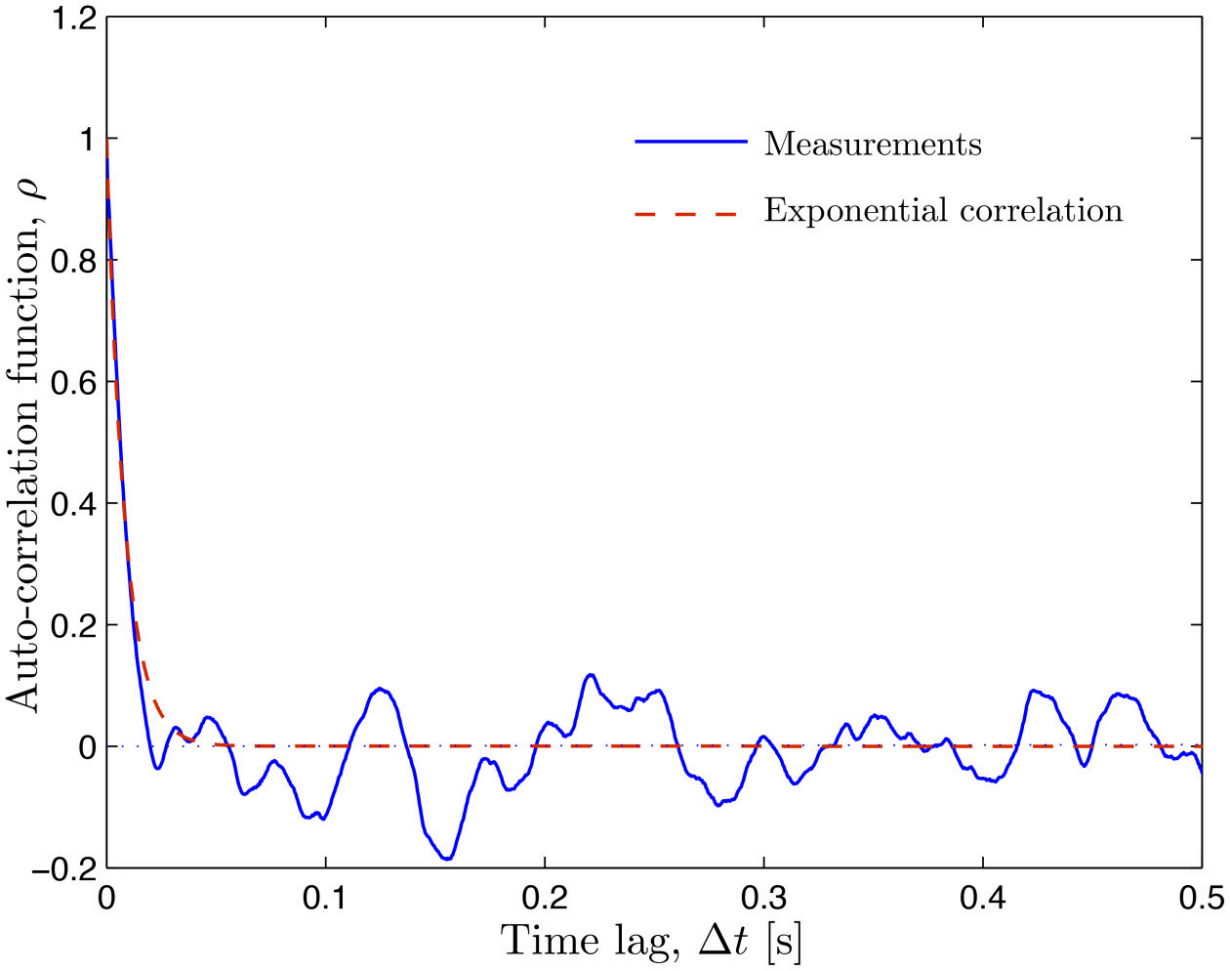
**Auto-correlation of the temporally fluctuating CFL width ***w*** reported by Ong et al. (2011a) (solid line) and the fitted exponential correlation function ρ(Δ***t***) = exp(−Δ***t***∕***l***_***t***_) with the correlation length ***l***_***t***_ = 0.007 s (dashed line)**.

The CFL width measurements (Ong et al., 2011a) are used to construct a probabilistic model for the random input parameter *w*(θ, *t*; ω) = *r*_2_ − *r*_1_ in which the random RBC-CFL interface *r*_1_(θ, *t*; ω) is given by Equation (11). These data, which represent temporal fluctuations of *w* at a single spatial location (say, θ = 0), give rise to the histogram and auto-correlation reported in Figures 1 and 2, respectively. This histogram (and the obvious fact that the CFL width is both non-negative and smaller than the vessel radius *r*_2_) indicates that the random field *w*(θ, *t*; ω) is non-Gaussian. We fit the histogram in Figure 1 with a beta distribution $f_{w}\left(W\right)=B^{-1}r_{2}^{1-\alpha -\beta }W^{\alpha -1}{\left(r_{2}-W\right)}^{\beta -1}$, where *B*(α, β) = Γ(α + β)∕[Γ(α)Γ(β)] is the beta function, Γ(·) is the complete gamma function, 0 ≤ *W* ≤ *r*_2_, and α > 0 and β > 0 are shape parameters. Setting α = 4.358 and β = 32.9 provides the best data fit, resulting in the mean CFL width $\bar{w}=2.73$ μm. The auto-correlation data in Figure 2 were fitted with an exponential correlation function $\rho \left(t,t^{\prime }\right)=\exp\left(-|t-t^{\prime }|∕l_{t}\right)$, yielding the correlation length *l*_*t*_ = 0.007 s.

**Figure 3:**
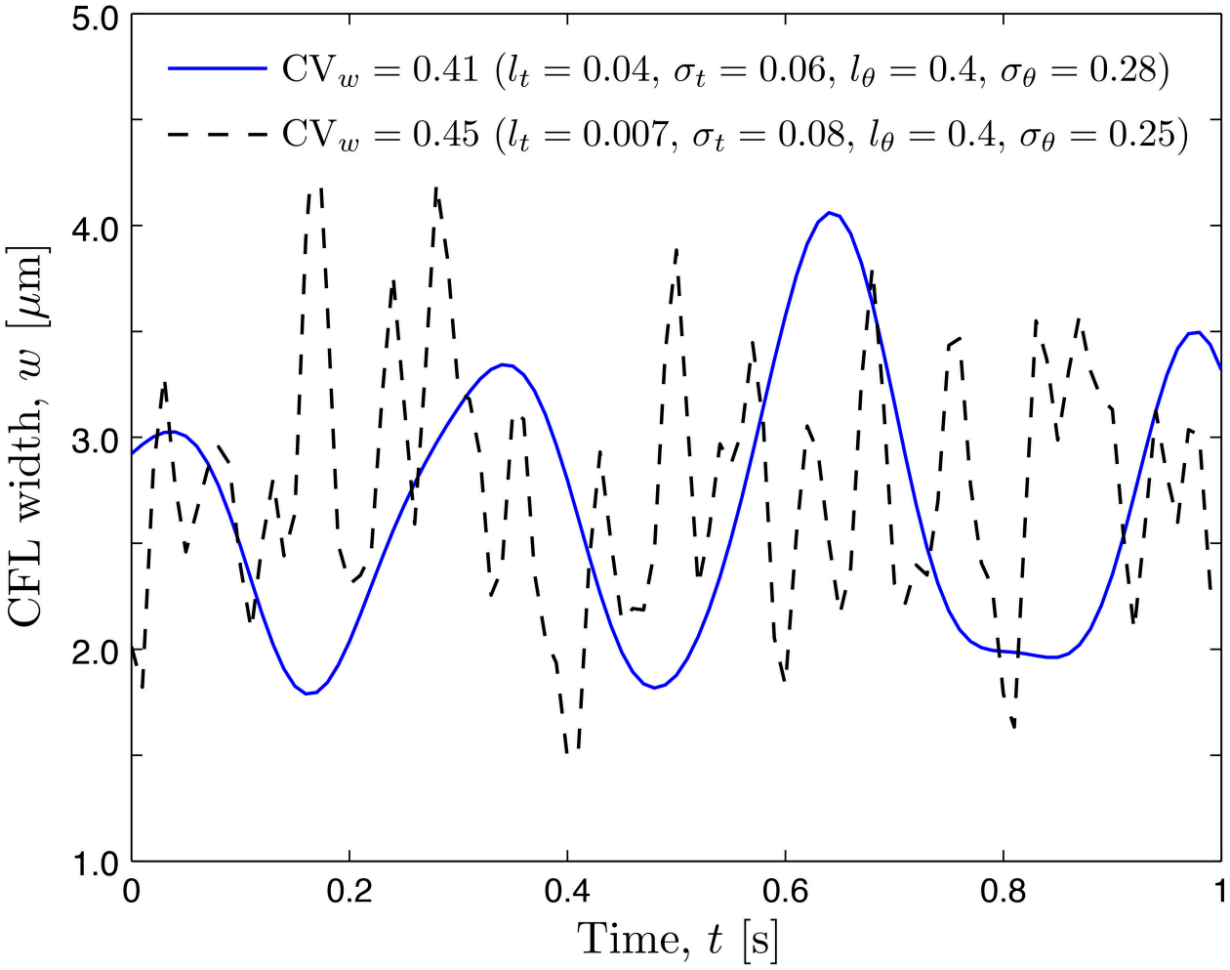
**Realizations of temporal fluctuations of the CFL width ***w*** at angular coordinate θ = 0.0 corresponding to two sets of statistical parameters**.

Experimental limitations preclude data acquisition at multiple azimuths θ, which requires us to postulate a probabilistic model for *r*_θ_(θ; ω). In analogy with its temporal counterpart *r*_*t*_(*t*; ω), we chose *r*_θ_(θ; ω) to have the beta distribution with unit mean and variance $\sigma_{\theta }^{2}$ and the exponential correlation function with correlation length *l*_θ_. In the formulation provided by Equation (12), the amplitude of spatio-temporal (in the angular coordinate θ and time *t*) fluctuations of both the RBC-CFL interface *r*_1_(θ, *t*; ω) and CFL width *w*(θ, *t*; ω) = *r*_2_ − *r*_1_ increases with the variances $\sigma_{\theta }^{2}$ and $\sigma_{t}^{2}$, while the smoothness of these fluctuations increases with the correlation lengths *l*_θ_ and *l*_*t*_. This behavior, which reflects chaotic motion of RBCs in the blood core, is demonstrated by two representative realizations of the random CFL width shown in Figure 3.

### 3.2. Random fluctuations of wall shear stress

The CFL width *w* in Equation (10) is inversely proportional to the wall shear stress (WSS) τ_*w*_. Hence the random spatio-temporal fluctuations in *w* induce corresponding fluctuations in τ_*w*_. In the computations of the WSS we use the values of the edge velocity *V*_*e*_ = 0.54 mm/s and the corresponding pressure gradient *J* = 2.15 × 10^4^ computed by fitting the smooth-wall model to the experimentally observed peak NO concentration of 11.2 nM.

**Figure 4:**
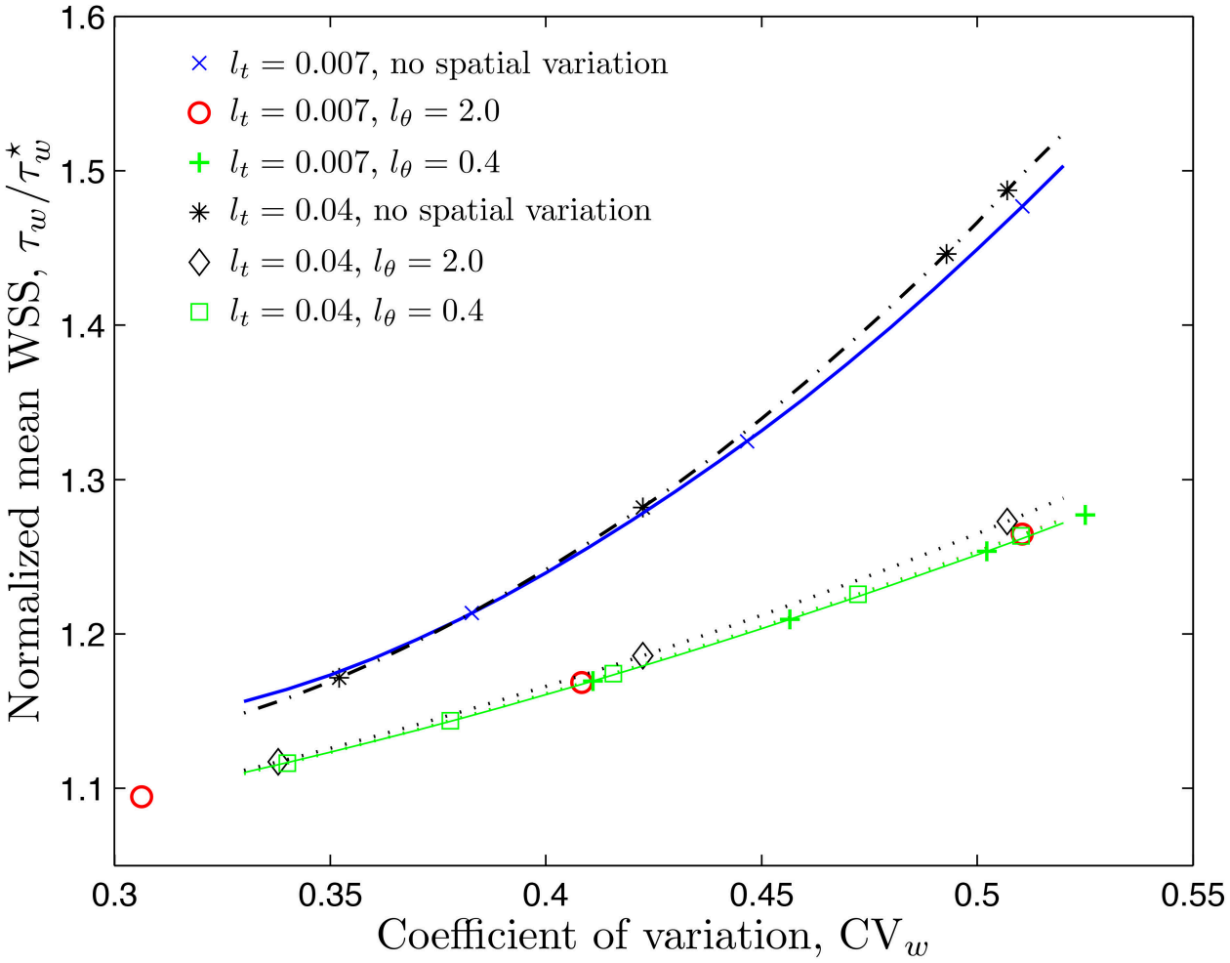
**Mean WSS, normalized with the smooth-vessel WSS $\tau_{w}^{⋆}$, (and mean NO production rate, normalized with the control production rate ${\dot{q}}^{⋆}$) as a function of the coefficient of variation (${\text{CV}}_{w}=\sigma_{w}/\bar{w}$) of the CFL width for several combinations of constitutive statistical parameters**.

The statistic commonly available from experimental studies similar to Kim et al. (2006, 2007) and Ong et al. (2011a) is the coefficient of variation of the CFL width, ${CV}_{w}=\sigma_{w}∕\bar{w}$. Figure 4 shows how the mean WSS ${\bar{\tau }}_{w}$, normalized with the smooth-vessel smooth-vessel WSS $\tau_{w}^{⋆}$, increases with CV_*w*_. (Recall that the fixed/smooth boundaries of the CFL correspond to CV_*w*_ = 0 and ${\bar{\tau }}_{w}∕\tau_{w}^{⋆}=1$). The rate of growth of the mean WSS depends on the model's statistical parameters, some of which, especially *l*_θ_, are not found in the experiments (Kim et al., 2006, 2007; Ong et al., 2011a). Fortunately, Figure 4 reveals that the mean WSS is nearly insensitive to *l*_θ_, being dominated by the temporal fluctuations statistics that are more readily measurable.

### 3.3. NO production rate

It follows from Equation (10) that the rate of NO production by the endothelium, ${\dot{q}}_{2}$, is directly proportional to the WSS. When normalized by the control production rate ${\dot{q}}^{⋆}$, it is equal to the ratio $\tau_{w}∕\tau_{w}^{⋆}$. In other words, the statistics of the ratios ${\dot{q}}_{2}∕{\dot{q}}^{⋆}$ and $\tau_{w}∕\tau_{w}^{⋆}$ coincide. Therefore, Figure 4 also demonstrates how the mean NO production rate by the endothelium, ${\overset{{}^{\circ}}{\bar{q}}}_{2}∕{\dot{q}}^{⋆}$, increases with the coefficient of variation of the CFL width, *CV*_*w*_.

### 3.4. Mean profiles of NO concentration

Unless specified otherwise, the results reported below correspond to the hematocrit-dependent reaction rate *k*_1_ in Equation (4) given by the constitutive model in Equations (8) and (9). We start by computing a (deterministic) reference NO concentration $c_{NO}^{⋆}\left(r\right)$ as a solution of Equations (1)–(3) with smooth (constant) interfaces *r*_1_ and *r*_2_. It serves as an initial condition for transient stochastic simulations.

**Figure 5:**
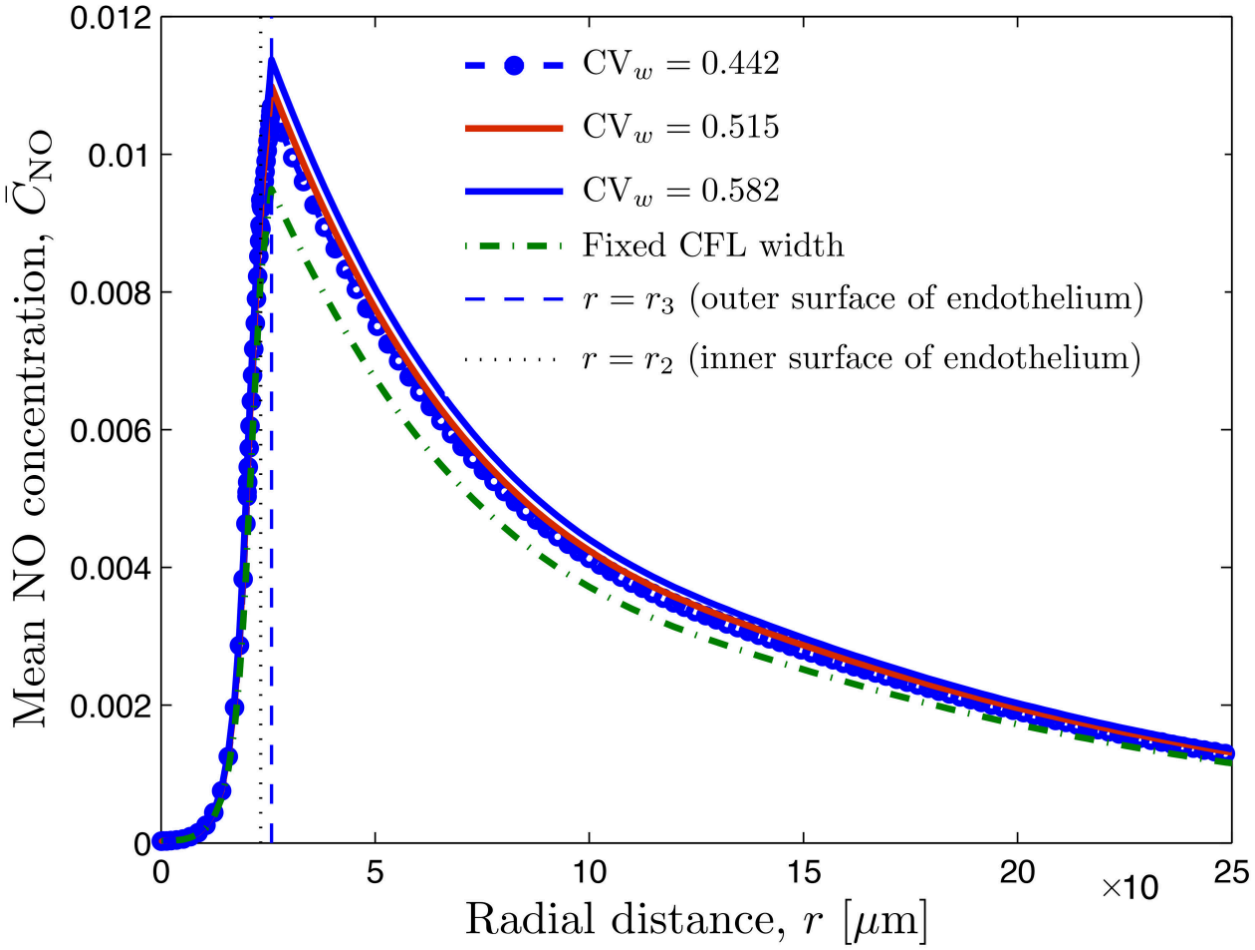
**Radial profile of mean NO concentration for several degrees of spatio-temporal variability of CFL quantified by CV_***w***_ = 0.442 (***l***_θ_ = 0.0 and σ_θ_ = 0.0), CV_***w***_ = 0.515 (***l***_θ_ = 1.0 and σ_θ_ = 0.07), and CV_***w***_ = 0.582 (***l***_θ_ = 1.0 and σ_θ_ = 0.1); in all three cases, ***l***_***t***_ = 0.008 and σ_***t***_ = 2.03**. Also shown is NO concentration corresponding to constant uniform CFL width. The vertical lines indicate the inner and outer surfaces of the endothelium.

The mean concentration profiles computed with these simulations, ${\bar{C}}_{NO}\left(r\right)$, are exhibited in Figure 5. While the NO production rates (${\dot{q}}_{2}$ and ${\dot{q}}_{3}$) on both sides of the endothelium (*r* = *r*_2_ and *r*_3_) are the same, the NO scavenging rate in the RBC core (0 ≤ *r* ≤ *r*_1_) is higher than that in the muscle tissue (*r* > *r*_3_). That is why the peak NO concentration is at the endothelium surface facing the tissue (*r* = *r*_3_).

Figure 5 also reveals that random fluctuations of the CFL width increase the NO availability relative to that predicted by the model that ignores them. This is to be expected, since these fluctuations enhance the NO production by the endothelium (Figure 4). NO production and availability increase with the the degree of roughness of the random RBC-CFL interface *r*_1_(θ, *t*; ω): the higher CV_*w*_ and/or the smaller the correlation lengths *l*_*t*_ and *l*_θ_, the rougher the interface is.

The simulation results reported in Table 2 demonstrate the relative importance of temporal and angular fluctuations of the CFL width on NO availability. The latter is reported in terms of the ratio of the peak NO concentrations, $R=({\bar{C}}_{\text{max}}-C_{\text{max}}^{⋆})/C_{\text{max}}^{⋆}$, where $C_{max}^{⋆}=C_{NO}^{⋆}\left(r_{3}\right)$ and ${\bar{C}}_{max}={\bar{C}}_{NO}\left(r_{3}\right)$. Larger values of R indicate stronger impact of the CFL width fluctuations.

### 3.5. Effect of constitutive models

The above-made estimates of NO production and availability rely on the NO scavenging rate *k*_1_(*t*; ω) given by the constitutive law in Equations (8), (9), which treats blood as a single-phase fluid. The alternative constitutive model for *k*_1_(*t*; ω), which explicitly accounts for the CFL presence, is given by Equations (6) and (7). Our simulations demonstrate that the difference between the mean peak NO concentrations predicted with the two models is less than 1% (Table 2). This provides a confirmation of the robustness of our predictions of expected NO production and availability with respect to model selection for the scavenging rate.

### 3.6. Effect of dextran infusion

In the experiments reported by Ong et al. (2011a), infusion of a plasma expander dextran increases the average CFL width from $\bar{w}=2.73$ to 3.22 μm. It also enhances fluctuations of the CFL width, increasing *CV*_*w*_ from 0.443 to 0.509 while leaving the correlation length *l*_*t*_ practically unchanged (it increases from 0.007 s before the dextran infusion to 0.008 s after). To match the decrease in the reported peak NO concentration from 11.2 to 9.5 nM, we recalculated the value of the pressure gradient *J* = 2.15 × 10^4^ and 1.72 × 10^4^.

**Figure 6:**
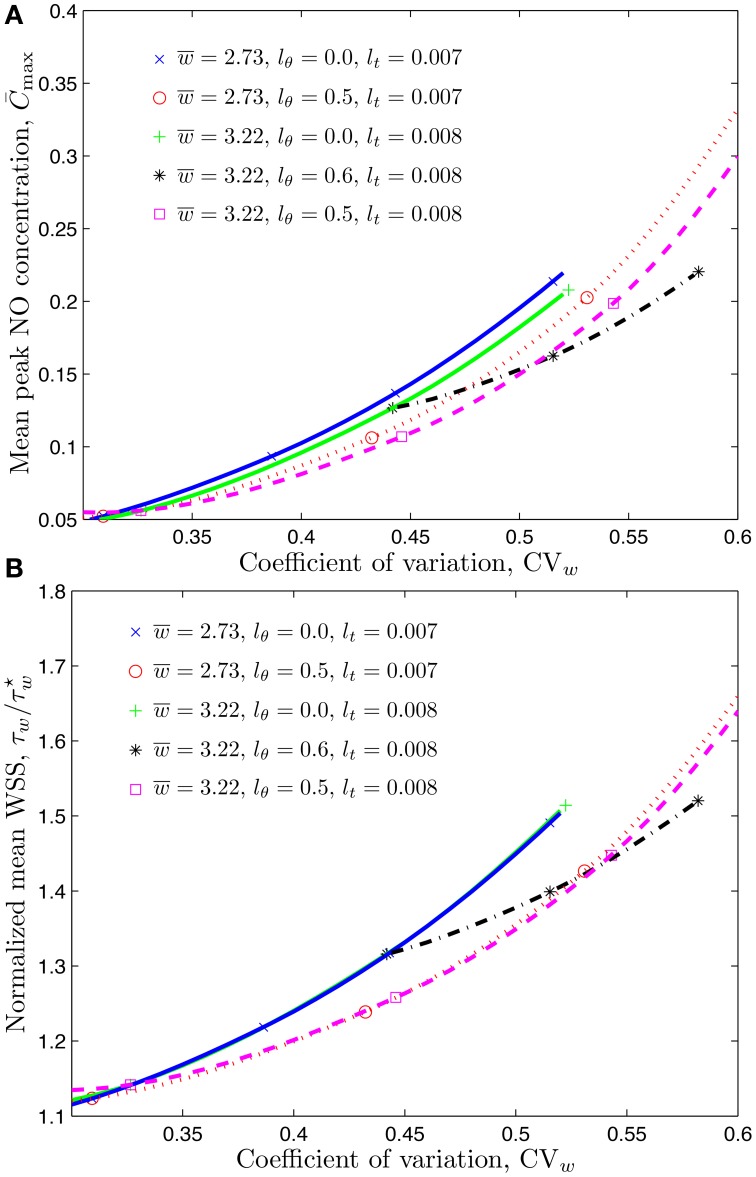
**Mean peak NO concentration (A) and mean WSS ratio (B) corresponding to temporal and spatial variations of CFL whose mean value is $\bar{w}=2.73$ μm or 3.22 μm**.

Figure 6 demonstrates that changing the mean CFL width (from 2.73 to 3.22 μm) does not significantly change the mean WSS, but has a more pronounced effect on the mean peak NO concentrations. The peak NO concentration ratio R reported in Table 2 further emphasizes this effect.

## 4. Discussion

We developed a computational framework to quantify the impact of spatio-temporal fluctuations in the CFL width on the production and transport of NO. This is accomplished by treating the RBC-CFL interface (and the corresponding CFL width) as a space-time correlated random field. This surface is represented via Karhunen-Loéve and Fourier expansions. The differential equations describing blood flow and NO production and transport, defined on random simulation domains, were solved by using a stochastic collocation method.

Our analysis demonstrates that the two-phase nature of microcirculatory flow, partitioned between a central blood column and a peripheral cell free plasma layer, causes stochastic flow variability in the CFL that influences NO bioavailability. Our findings are qualitatively comparable to those obtained with the deterministic analysis conducted by Ong et al. (2011a). However, they differ in an important way since the statical parameters on which they are based can be used to predict NO production, as well as other effects related to the variability of the CFL, with data from other experiments where the same statistical properties can be assessed.

Flow variability has a homeostatic role in the microcirculation where it is a factor in the control of blood flow and inflammation through biochemical mechanotransduction modulation of the production of NO and prostaglandins. Large Reynolds numbers and flow variability in the central circulation can cause the up-regulation of virtually all atherogenic or pro-inflammatory genes ultimately promoting the development of atherosclerotic plaques (Cabrales et al., 2011). Experimentally it is shown that a principal effect of flow (and shear stress) variability is the up and down regulation of the expression of a multitude of genes in endothelial cell cultures (Yee et al., 2008).

The rate of production of NO by mechanotransduction differs between steady and time-dependent flows, the latter resulting in a significantly higher production of NO. This phenomenon has been evidenced in studies using horizontal parallel plates flow chambers in which endothelial cells grow to a confluent layer (Ruel et al., 1995). Continuous changes of flow or “ramp” flow (Frangos et al., 1996) and step changes (Andrews et al., 2010) yielded significantly greater NO production than constant steady flow. Experimental studies also show that the rate of NO production is higher for sinusoidal flow vs. steady flow with the same mean (Noris et al., 1995; Li et al., 2005). There is evidence that flow variation frequency of about 1 Hz, independent of shear, is a determinant of the endothelial responses to pulsatile flow (Balcells et al., 2005).

Although vessel wall shear rates and shear stress (WSS) are similar throughout the circulation, as proposed by the “uniform shear stress hypothesis” (Kassab and Fung, 1995), flow variability is quite different. The main source of flow variability is the periodic action of the heart, which can also cause flow instabilities in locations with large Reynolds numbers. This variability decreases from the systemic blood vessels to the microcirculation where it is attenuated to an amplitude of 1–2% of mean flow (Intaglietta et al., 1971) in the arterioles.

Blood flow in microvessels also undergoes periodic flow changes whose amplitude can reach 100% of mean flow due to the phenomenon of vasomotion. This activity has fundamental frequency of the order of minutes for the larger arterioles, increasing as vessel size decreases (Colantuoni et al., 1984). This phenomenon has received many interpretations since it has been found in some normal conditions but not all, and is elicited by ischemic and low pressure states (Schmidt et al., 1992).

Our analysis and results show the importance of a microscopic random flow variability, whose primary effect is to increase the bioavailability of NO at the vessel/CFL interface. This variability has two components: a vessel wall component due to the unevenness of the endothelium (Barbee et al., 1994; Barbee, 2002) and the blood/CFL interface component. The latter is absent in experimental studies of cell culture flow chambers perfused with culture media, i.e., in the majority of studies.

There is presently no specific evidence of the effect on mediator production rate by stochastically induced mechanotransduction. However, since random excitation contains all the spectral components of sinusoidal, ramp and step flow variability it is likely that random excitation increases the rate of mediator production. These effects are largely unexplored, since endothelial mechanotransduction has been primarily studied with non-blood, steady or varying flow conditions on a scale that is macroscopic relative to the dimensions of endothelial cells (Chien, 2007). Yet, cell-scale variations of shear stress can cause large shear stress gradients (Davies et al., 2003; Leiderman et al., 2008). Experimental studies to determine the physiological role of this variability are not available.

Stochastic variability of WSS is fundamentally distinct from the temporal variability studied in cell cultures. The latter is macroscopic and has primarily a local effect in that it is absent in the microcirculation, where temporal flow changes occur in a time frame of minutes to hours, a condition of quasi steady state. The stochastic fluctuations of the CFL width generate a persistent time-dependent microscopic component of WSS, which is not tested by current experimental methods. Our results and these considerations suggest that endothelial flow chamber experiments aimed at understating the consequences of mechanotransduction on cardiovascular regulation should include the conditions that preserve the stochastic nature of the blood flow/microvessel wall interaction, i.e., use blood as the flow medium. This appears to be the only significant source of time-dependent variability of shear stress in the microcirculation. It may be significantly affected by changes in the composition of either blood (due to hemorrhage and anemia) or plasma (possibly due to diabetes and hypertension).

## Author contributions

SP carried out numerical implementation of the proposed algorithms, participated in the design of the study and drafted the manuscript; MI formulated the physiological problem, participated in the design of the study, and helped draft the manuscript; DT designed and coordinated the study, and helped draft the manuscript. All authors gave final approval for publication.

## Funding

This research was supported in part by NIH under award numbers R01-HL064395 and R24-HL64395, Air Force Office of Scientific Research under award number DE-FG02-07ER25815, and by National Science Foundation under award number DMS-1522799.

### Conflict of interest statement

The authors declare that the research was conducted in the absence of any commercial or financial relationships that could be construed as a potential conflict of interest.

## Appendix A

### Transformed transport equations

A general transformation of coordinates ξ_*i*_(*x*_1_, *x*_2_) transforms the Fickian flux **F**(**x**) = −*D*∇_**x**_*C*_NO_ into a vector **F**(**ξ**) whose contravariant components *F*^1^ and *F*^2^ are given by

(A1) $$\begin{array}{l} F^{i}=D\frac{1}{J}\overset{2}{\underset{k=1}{\sum }}A_{ik}\frac{\partial C_{NO}}{\partial \xi_{k}},\;i=1,2 \end{array}$$

where *J* is the transformation Jacobian in Equation 20 and

(A2a) $$\begin{array}{lll} A_{11}= & {\left(\frac{\partial x_{2}}{\partial \xi_{2}}\right)}^{2}\;+\;{\left(\frac{\partial x_{1}}{\partial \xi_{2}}\right)}^{2},\; & \; \end{array}$$

(A2b) $$\begin{array}{lll} A_{12}= & A_{21}=-\frac{\partial x_{2}}{\partial \xi_{1}}\frac{\partial x_{2}}{\partial \xi_{2}}-\frac{\partial x_{1}}{\partial \xi_{1}}\frac{\partial x_{1}}{\partial \xi_{2}},\; & \; \end{array}$$

(A2c) $$\begin{array}{lll} A_{22}= & {\left(\frac{\partial x_{2}}{\partial \xi_{1}}\right)}^{2}+{\left(\frac{\partial x_{1}}{\partial \xi_{1}}\right)}^{2}.\; & \; \end{array}$$

Furthermore,

(A3) $$\begin{array}{ll} \nabla_{\text{x}}\cdot \text{F}=\frac{1}{J}\left(\frac{\partial F^{1}}{\partial \xi_{1}}+\frac{\partial F^{2}}{\partial \xi_{2}}\right)\equiv \frac{1}{J}\nabla_{\xi }\cdot \text{F}. & \; \end{array}$$

Substituting Equations (A1)–(A3) into Equation (1) leads to Equation (21).

## Appendix B

### Numerical implementation

Let ${\left\{{\text{Ψ}}_{m}\left(\text{Y}\right)\right\}}_{m=0}^{M}$ denote a set of multidimensional orthogonal polynomials of the random vector $\text{Y}\left(\omega \right)\equiv {\left(Y_{1},\ldots ,Y_{K}\right)}^{T}$. The polynomials are chosen to have the ensemble means ${\bar{\text{Ψ}}}_{0}=1$ and ${\bar{\text{Ψ}}}_{k}=0$ (*k* ≥ 1) and to satisfy the orthogonality condition $\langle {\text{Ψ}}_{i}{\text{Ψ}}_{j}\rangle =\langle {\text{Ψ}}_{i}^{2}\rangle \delta_{ij}$, where the 〈·〉 operator is defined by

(A4) $$\begin{array}{ll} \langle {\text{Ψ}}_{i}{\text{Ψ}}_{j}\rangle \equiv \int {\text{Ψ}}_{i}\left(\text{Y}\right){\text{Ψ}}_{j}\left(\text{Y}\right)W\left(\text{Y}\right)dY_{1}\ldots dY_{K}, & \; \end{array}$$

δ_*ij*_ is the Kronecker delta, and *W*(**Y**) is a weight function corresponding to a given polynomial type. The size of the polynomial set, *M*, is determined by the “stochastic dimension” *K* and the order *P* of polynomials Ψ_*k*_, according to

(A5) $$\begin{array}{l} M=\frac{\left(K+P\right)!}{K!P!}\;-\;1. \end{array}$$

Polynomial chaos expansions represent a system state, e.g., *u*(**ξ**, ω), a random field whose ensemble statistics are to be determined, as a series

(A6) $$\begin{array}{ll} u\left(\xi ,\omega \right)=\overset{M}{\underset{k=0}{\sum }}{û}_{k}\left(\xi \right){\text{Ψ}}_{k}\left[\text{Y}\left(\omega \right)\right]. & \; \end{array}$$

Instead of stochastic Galerkin method, we adopt a stochastic collocation method to solve the random boundary problem. This method is described below.

## Appendix C

### Stochastic collocation method

Consider a stochastic partial differential equation

(A7) L(x,ξ(ω);u) = f(x,ξ(ω)).

Its solution is approximated by using a Lagrange formula

(A8) $$\begin{array}{ll} u\left(\text{x},\xi \left(\omega \right)\right)=\overset{Nq}{\underset{k=0}{\sum }}u_{k}\left(\text{x},\xi^{k}\right)L_{k}\left(\xi \right), & \; \end{array}$$

where $u_{k}\left(\text{x},\xi^{k}\right)$ is the solution at the set of collocation points ${\left\{\xi^{k}\right\}}_{k=0}^{N_{q}}$, *L*_*k*_ is the Lagrange polynomial of order *N*_*q*_ + 1, and *L*_*i*_(**ξ**_*j*_) = δ_*ij*_. Next, Equation (A8) is substituted into the inner product formula given by Equation (A4) with the basis functions chosen to be Dirac delta functions δ(**ξ** − **ξ**^*k*^). Then *N*_*q*_ + 1 uncoupled deterministic problems are solved at the collocation points,

(A9) $$L(x,\xi^{k};u_{k})=f(x,\xi^{k}),\text{ for }k=0,\ldots ,N_{q}.$$

Once the solutions at a set of collocation points are obtained, their statistics are computed by using the corresponding quadrature rule,

(A10) $$\begin{array}{ll} \bar{u}\left(\text{x}\right)=\int_{\Gamma }u\left(\text{x},\xi \right)p\left(\xi \right)d\xi \approx \overset{Nq}{\underset{k=1}{\sum }}u\left(\text{x},\xi^{k}\right)w_{k} & \; \end{array}$$

and

(A11) $$\begin{array}{ll} \sigma_{u}^{2}\left(\text{x}\right)=\int_{\Gamma }{\left[u^{\prime }\left(\text{x},\xi \right)\right]}^{2}p\left(\xi \right)d\xi \approx \overset{Nq}{\underset{k=1}{\sum }}u{\left(\text{x},\xi^{k}\right)}^{2}w_{k}-{\bar{u}}^{2} & \; \end{array}$$

where ${\left\{w_{k}\right\}}_{k=1}^{Nq}$ is a set of weights corresponding to the set of quadrature points ${\left\{\xi^{k}\right\}}_{k=0}^{Nq}$.
